# Predicting the process of extinction in experimental microcosms and accounting for interspecific interactions in single-species time series

**DOI:** 10.1111/ele.12227

**Published:** 2013-12-05

**Authors:** Jake M Ferguson, José M Ponciano

**Affiliations:** Department of Biology, University of FloridaGainesville, FL, USA

**Keywords:** Autocorrelation, community effects, extinction, first passage time, moving-average model, PVA, time series

## Abstract

Predicting population extinction risk is a fundamental application of ecological theory to the practice of conservation biology. Here, we compared the prediction performance of a wide array of stochastic, population dynamics models against direct observations of the extinction process from an extensive experimental data set. By varying a series of biological and statistical assumptions in the proposed models, we were able to identify the assumptions that affected predictions about population extinction. We also show how certain autocorrelation structures can emerge due to interspecific interactions, and that accounting for the stochastic effect of these interactions can improve predictions of the extinction process. We conclude that it is possible to account for the stochastic effects of community interactions on extinction when using single-species time series.

## Introduction

One of the most important modelling applications in conservation ecology is predicting future population abundances. The practice of population viability analysis (PVA) connects stochastic population models to data and is used to understand the factors limiting population growth and to assess risk of extinction or falling beneath a certain abundance (e.g. Shaffer 1981; Staples *et al*. 2005). Despite a body of well-developed theory on population regulation, there has been relatively little work validating quantitative predictions of PVA models and methods (but see attempts by Brook *et al*. 2000; Lindenmayer *et al*. 2003). This is of particular importance for species of conservation concern where little data may be in hand regarding life-history details and knowledge of the underlying biological processes is limited. In these cases, ecologists often rely on very basic populations models of growth, density dependence, and variability to make predictions.

Characterising the extinction process has been an important goal for theoretical ecology (Lande & Orzack 1988; Dennis *et al*. 1991; Foley 1994). Classic results have provided useful scaling laws for the time to extinction (e.g. Ludwig 1976; Leigh 1981), rules of thumb for dealing with the types of variation that can drive populations to extinction (e.g. Boyce 1992; Lande 1993); as well as the effects of age (Lande & Orzack 1988) and spatial structure (e.g. Holt 1985; Hanski & Gilpin 1997) on extinction. Despite the broad scope of current extinction theory, determining the impacts of community interactions on extinction risk remains an elusive task. Although a small handful of PVA studies have taken a multispecies approach when assessing population viability (as reviewed in Sabo 2008), we know of no work that has attempted to explicitly incorporate the effects of interspecific interactions on extinction when only single-species time-series data are available. Thus, developing modelling methods that include these effects is an important step in extending the application of extinction theory to real ecological systems where available data are often limited.

Microcosm experiments can facilitate the essential task of anchoring theoretical predictions with experimental observations. Although microcosm experiments have been argued to greatly oversimplify ecological systems (Carpenter 1996), they also offer a test bed for theory that is unambiguous. Past experimental tests of extinction have primarily focused on testing qualitative differences between predictions, rather than quantitative predictions, thus potentially limiting their applicability (Griffen & Drake 2008).

It is not clear whether standard models of population variability will adequately account for ecological interactions, such as predation and competition, as these interactions are likely to induce structured variation (Royama 1981; Boyce 1992; Stenseth *et al*. 1998; Abbott *et al*. 2009). Here, we examined the ability of a suite of unstructured and structured variance models to predict extinction times observed in an experimental microcosms of *Daphnia pulicaria*. At the same time we assessed the effects of a number of common model assumptions, including the particular form of density dependence, the model for the variance of the growth rate and the transition probability distribution of the discrete growth process. Importantly, the nature of the experimental data sets that we used allowed us to test the ability of these model variations to predict the extinction process for two different classes of dynamics often dealt with in PVA analyses. In the first set of experiments, the populations fluctuate around a steady state, whereas in the second experiment population tends to decline over time. Thus, the scenarios tested allow our results to be extended beyond this study.

## Models & Methods

### Experimental data

**Figure 1:**
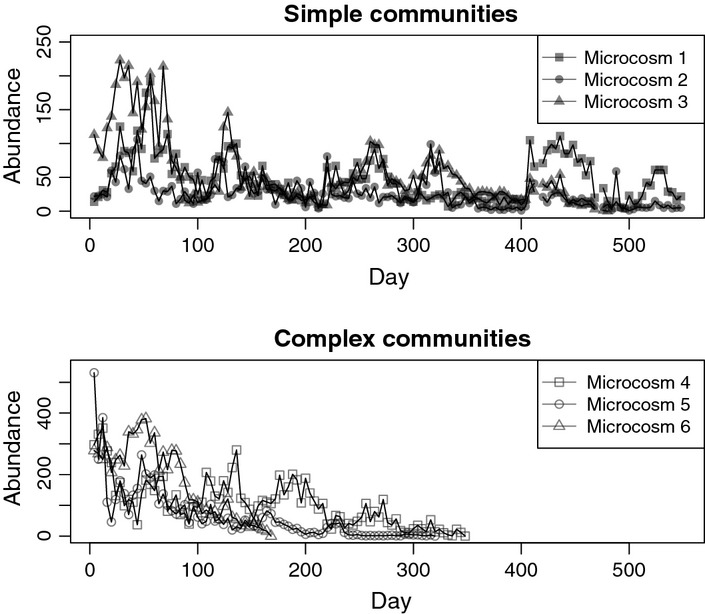
Population time series for replicates of different experimental conditions. Simple communities included a consumer *Daphnia pulicaria* and planktonic resource. Complex communities included the consumer and resource along with competitors.

The data sets re-analysed in this study come from previous work by Grover *et al*. (2000) who followed experimental populations of *D. pulicaria* for nearly 2 years (Fig. 1). The original experiment discarded the first 140 days of data to exclude the initial transient phase of population dynamics and we followed the same practice. We considered two of their experimental treatments where each treatment had three replicates. In one treatment, population abundances fluctuated around the steady state, displaying quasi-stationary dynamics, whereas in the other treatment abundances displayed a decline towards extinction. These different dynamics correspond to the small-population paradigm and the declining-population paradigm as defined by Caughley (1994). Both scenarios are of interest for management and conservation purposes.

The experimental conditions consisted of three replicates of simple community microcosms and three replicates of complex community microcosms. Simple communities were composed of consumer–resource interactions between *D. pulicaria* feeding on green algae (*Scenedesmus acutus*, *Scenedesmus quadricauda* and *Chlorella sp*.) and other indigenous microorganisms, whereas complex communities were composed of the consumer–resource system plus additional grazers (*Simocephalus vetulus* and *Cypridopsis obesa*). While *D. pulicaria* and the *S. vetulus* are filter feeders and have been shown to compete in microcosm experiments (Frank 1952), *C. obesa* is a scraper (Roca *et al*. 1993). Sampling occurred every 4 days. In each sample approximately 58% of the microcosm was recorded by video-camera and animals were counted. *D. pulicaria* display a generation time of 10–50 days at the temperature used (15^°^Celsius). More experimental details are provided in Grover *et al*. (2000).

### Population models

#### Single-species models

Our analysis was based on a derivation for the mean and variance of a discrete-time abundance model with demographic and environmental variance. A full derivation of the mean and variance for abundances is provided in Appendix A, although some details are presented here. Throughout the text we use the convention that random variables are denoted using capital letters, such as *N*_*t*_, whereas realisations of the random variable are lower case (e.g. *n*_*t*_). In what follows, *N*_*t*_ denotes the random population abundances at time *t*.

We start by assuming that every individual gives birth on average to *λ* offspring per generation with a variance given by *ϕ*^2^, the demographic variation in reproduction (see Appendix A for more details regarding the assumptions of this variance model). Next, we assume that *λ* can vary randomly over time with variance given by *φ*^2^, the environmental variation in reproduction. After reproduction offspring and parents survive with probability *p*_*t*−1_ = *p*_0_*p*(*n*_*t*−1_), where *p*_0_ is the density-independent survival and *p*(*n*_*t*−1_) is the density-dependent survival. We note that the individual parameters of survival and reproduction in the product *λp*_0_ are non-identifiable (as shown in Appendix A), therefore we define *r* = *λp*_0_. Following these assumptions, the expected value and variance for current abundances as a function of the previously observed abundances are as follows:


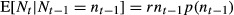
(1)


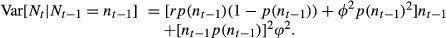
(2)

The first term in eqn (2) is a factor of *n*_*t*−1_ and is referred to as the demographic stochasticity. This expression comes from computing the average variability in the reproduction and survival process (see Appendix A). Thus, this variance component corresponds to the overall contribution of demographic variance to the total population variance, always scaling like *n*_*t*−1_ regardless of the particular form of the reproduction and survival. Note that only in the case of density independence, when *p*(*n*_*t*−1_) = 1, the demographic variance of the population process is exactly proportional to *n*_*t*−1_. When survival is density dependent, the demographic stochasticity will scale as a more complex function of *n*_*t*−1_ (Saether *et al*. 1998; Drake 2005).

The second half of eqn (2) scales by 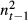 and is referred to as the environmental stochasticity. This term comes from computing the variability over time of the average number of offspring produced that survive to the next generation (Appendix A). This explicitly accounts for temporal fluctuations in the reproduction process, thus translating the concept of the environmental variability into an analytical variance component of the population growth process.

#### Interspecific interaction models

Previous work has shown that autocorrelation can be induced through population interactions that are subjected to variability (Royama 1981; Abbott *et al*. 2009). A simple model of two interacting species on the log-scale (*X*(*t*) ≡  ln *N*_*t*_) can be written as:


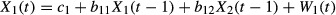
(3)


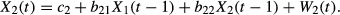
(4)

The *b*_11_ and *b*_22_ terms correspond to density-dependence regulation terms, the *b*_12_ and *b*_21_ terms correspond to interactions between life-history stages or species and the *W*(*t*) terms represent demographic and environmental stochasticity. This system can be viewed as a linearised approximation to more complex functional responses (Ives *et al*. 2003). The system of equations (eqns (3) and (4)) can be re-expressed as a univariate autoregressive- moving-average (ARMA) model for the species of interest, either *X*_1_(*t*) or *X*_2_(*t*), obtaining an ARMA(2,1) model (see Appendix B for derivation). In this work we refer to the AR(1) component as the density-dependence model, whereas we refer to the AR(2) component as simply an AR autocorrelation model, consistent with an extensive literature on the effects of environmental autocorrelation (e.g. Royama 1981). This AR(2) term also has been called the lagged density dependence by Turchin (1990).

This approach can be further generalised such that the univariate stochastic dynamics for a species in a community of *n* species will follow an ARMA(*n*, *n* − 1) model (Abbott *et al*. 2009). In the two species case, the magnitude of the autoregressive (AR) component is the difference between interspecific interactions and intraspecific interactions, *b*_12_*b*_21_−*b*_11_*b*_22_. The sign and magnitude of the AR parameter gives a measure of the balance between interspecific interactions (*b*_12_*b*_21_) and growth and intraspecific interactions (*b*_11_*b*_22_) in the system. In contrast, the moving-average (MA) component that is generated is a function of how well the interspecific interaction propagates perturbations. The MA parameter is maximised when the species interacting with the focal species follows a random walk, such that perturbations from equilibrium are propagated by the interacting species, rather than being dampened by a density-dependent response (Appendix B, eqn B5). In our model formulation, the autocorrelation components effect both the demographic and environmental variances, and we limit ourselves to fitting one AR component (in addition to the density dependence) and one MA component based on time-series diagnostics presented in Appendix C.

### Model comparisons

We considered a number of possible assumptions that might be made when conducting a PVA to construct a realistic but tractable set of models. The effects of each model assumption identified were assessed independently. These assumptions are as follows: (1) the form of the density dependence, (2) the distributional form of the transition pdf, (3) the form of the autocorrelation and (4) the variance model of the process error. For each of the assumptions, we considered a handful of explicit alternative specifications and evaluated the inferential consequences of considering each of these components, one at a time. Thus, four sets of independent comparisons were done. The comparisons are summarised in Table1.

**Table 1:** Fixed and varied components in model comparisons

	Comparison 1	Comparison 2	Comparison 3	Comparison 4
Density dependence	**Ricker**	Ricker	Ricker	Ricker
	**Beverton–Holt**			
	**Gompertz**			
	**Exponential**			
Transition distribution	Gamma	**LN**	Gamma	Gamma
		**NB**		
		**Gamma**		
Autocorrelation	None	None	**None**	None
			**AR**	
			**MA**	
			**ARMA**	
Variance	D + E	D + E	D + E	**D + E**
				**None**
				**E only**
				**D only**

For each model-set comparison we varied one assumption out of the four model components while fixing the others. Components that were varied are in bold. For entries that were fixed, we used the Ricker model of density dependence, the lognormal (LN) transition distribution and the demographic and environmental (D + E) model of stochasticity. Additional abbreviations used are negative binomial (NB), environmental variation only (E only) and demographic variation only (D only).

In comparison 1 (see Table1), we varied the model of density dependence while other components were fixed using functional forms for *p*(*n*_*t*−1_) in eqs (1) and (2a) that represent different hypotheses about the underlying population dynamics. These forms may be strictly interpreted as different hypotheses about population regulatory mechanisms (such as the form of interspecific competition), or as statistical models that approximate complex underlying population regulatory mechanisms. In any case, the form of density dependence controls the rate at which new individuals are added to the population as a function of abundance. We considered the overcompensatory Ricker (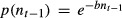) and logistic (*p*(*n*_*t*−1_) = 1 − *bn*_*t*−1_) models, as well as the undercompensatory Beverton–Holt (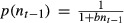) and Gompertz (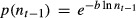) models. We also included the density-independent (*p*(*n*_*t*−1_) = 1) model of population growth as a ‘null hypothesis’. Although primarily associated with the mean response of the population, this term also affects the variance (eqn (2)). All forms of density dependence were required to be bounded between 0 and 1, therefore we implemented boundary constraints on parameters when necessary (e.g. for the logistic model). When comparing each of these density-dependence models, the transition distribution was assumed to be gamma and the variance to have demographic and environmental terms with no autocorrelation.

For comparison 2 (see Table1), we examined the properties of the transition pdf while other components were fixed. We tested the impact of the higher moments of a distribution and lattice (rounding) errors on predictions. Although the impact of the first two moments (the mean and variance) on extinction predictions has been studied using diffusion approximations (Ludwig 1976; Lande 1993) and simulations (Drake 2005; Melbourne & Hastings 2008), the impact of higher moments on predictions has not been considered. Thus, we varied the higher moments by fitting gamma, log-normal and negative binomial distributions to the data. Also, we tested for the presence of lattice effects by comparing predictions from the discrete-state, negative binomial distribution to gamma distribution, a similar continuous-state distribution. Lattice introduced in the approximation of an integer value variable by a continuous variable can appear in discrete-state models, and can have potential impacts on predictions (Henson *et al*. 2001). More details on how we matched the mean and variance of eqns (1) and (2) to the parameters of these distributions is provided in Appendix C. For the different transition pdf's in comparison 2, we assumed a Ricker model of density dependence and a demographic and environmental variance model with no autocorrelation.

In comparison 3 (see Table1) we tested the impact of the autocorrelation structure in the variance model while the other model components were fixed. Although eqn (2) is shown as conditional on *n*_*t*−1_, ecological interactions may induce other dependencies not properly captured by the density-dependence model. Both MA and AR autocorrelation models have been shown to capture information about interspecific and intraspecific interactions in ecological systems (Abbott *et al*. 2009). It has also been shown that MA models also arise due to measurement error (Dennis *et al*. 2006). AR and MA models can be combined into the ARMA correlation model to include effects of both models simultaneously (Shumway & Stoffer 2006). We incorporated AR, MA and ARMA correlation structures into the variance model and tested their impact on predictions of the extinction process. For all models in comparison 3, we assumed a gamma transition distribution, a Ricker model of density dependence and a demographic and environmental variance model.

For comparison 4 (see Table1), we examined several assumptions about the demographic and environmental terms in the variance model while fixing other model components. We tested whether purely demographic or environmental variance terms improved predictions over the full demographic and environmental model. The form in eqn (2) assumes that populations are subject to both demographic and environmental processes, an assumption that is not always made (e.g. Ellner & Holmes 2008). We also tested the impact of removing the density dependence in the demographic variance of the population growth rate *R*_*t*_ =  ln (*N*_*t*_/*N*_*t*−1_) similar to a test performed by Drake (2005). This led to the variance model 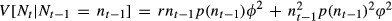. Under the comparison 4 model set, we fixed the transition distribution to be gamma, the form of density dependence to be Ricker and had no autocorrelations in the error structure.

### Parameter estimation

To fit the data we used the likelihood functions defined in Appendix C, where the joint probability of the observations is given by the product of the one-step transition distribution of the population process. The likelihoods of the transitions were constructed by matching the moments of eqns (1) and (2) to the mean and variances of the transition distribution (details provided in Appendix C). All models were fit to data using maximum likelihood estimation in the R statistical software environment (R Development Core Team 2012).

### First passage time simulations

After parameters were estimated, we used simulations to examine the consistency of our models with the observed extinction process. The metric we used to characterise extinction was the first passage time (fpt), defined as the time it takes for a population to first reach a quasi-extinction abundance *n* from some initial abundance *n*_0_. The distribution of fpts is denoted as 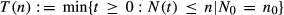 (Taylor & Karlin 1984). The likelihood of the fpt was calculated by determining the probability of obtaining the observed quantity *τ*(*n*) from the distribution of fpts predicted by model 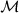, written as 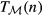, where our notation emphasises the fact that predictions are made conditional on a particular model. The *τ*(*n*)'s were obtained for all observed abundances, and 10^5^ simulations were used to obtain the distribution, 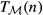, for each microcosm population. Because the fpt for these models is a discrete random variable, the probability of observing *τ*(*n*), 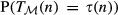, was set equal to the proportion of simulations which display a predicted fpt equal to the observed fpt. For the *i*th microcosm we used the likelihood of all *s*_*i*_ observed fpts for each community type. Because there were three microcoms per community type this likelihood is given by 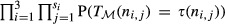 for all observed *n*_*i*,*j*_.

The fpt likelihoods for each model and each data set were used to calculate values of the Akaike information criterion (AIC), which were then used to select between models for each model-set comparison. The number of parameters, *k*, used in the AIC calculation was the number of parameters fit in the one-step transition likelihood. Models with lower AIC values were interpreted as doing a better job of explaining the extinction process. Using the FPT, rather than the raw abundances, can lead to different predictions as the FPT is a different summary of the data and may provide different information. We also calculated the BIC criterion for all models and found that all our conclusions were robust to the form of criterion used.

Finally, to obtain an absolute measure of the predictive error we calculated the root mean square error (RMSE), defined as 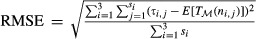. This measure gives an absolute measure of the error associated with the mean fpt predicted from model 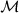, 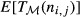, rather than a relative measure of evidence provided by AIC values.

### Measurement error

We tested the impact of measurement error on parameter estimates to determine if the observed MA parameter estimates were due purely to measurement error. The effects of measurement error were tested by simulating 100 time series using parameters from the Ricker density-dependent, demographic and environmental variance model with no autocorrelation for each of the three simple microcosm experiments. We rescaled the observations by 1/0.58 to account for the unobserved volume in each microcosm (Grover *et al*. 2000). A binomial sampling model was then applied to the simulated data where the probability of detection was 0.58. We estimated model parameters for the resulting observed time series, and the average and standard error of the moving-average parameter was calculated for each set of microcosm simulations. We compared these estimates to values observed from the microcosm experiments. If sampling error explained the moving-average parameter estimated in the simple community microcosms, then the observations should fall within approximately 2 standard errors from the estimated mean.

### Moving-average models

To test the potential impacts of non-measurement error MA processes on population persistence, we simulated abundance time series with parameters estimated from the model with Ricker density dependence, environmental and demographic stochasticity, and an MA autocorrelation model in microcosm 1. We then varied the MA parameter while keeping the other parameters fixed over 20 equally spaced values from −0.9 to 0.0. We calculated the mean time to extinction (MTE) by simulating until the time series reached 1 individual or less. We repeated this procedure 10 000 times to obtain stable estimates of the MTE.

## Results

### Model selection

**Figure 2:**
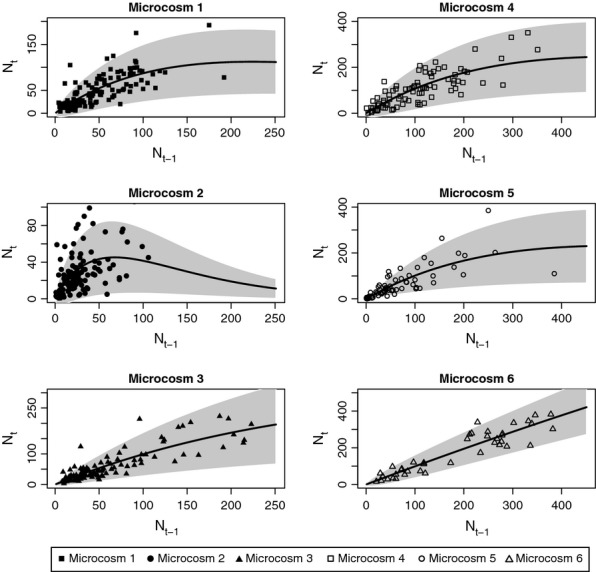
The mean population abundance for a Ricker model of density dependence (solid line) with demographic and environmental stochasticity, plotted for each observation (points) with the approximate 95% confidence intervals (grey area).

In the simple community experiment 2 of 3 populations persisted the entire duration of the experiment suggesting that these populations were stable, but subject to stochastic extinction events over longer timescales (Fig. 1). In contrast, all the complex community populations went extinct over the course of the experiment as abundances tended to decrease over time, suggesting that extinctions were due to deterministic processes. A model with Ricker density dependence and a gamma error structure containing both demographic and environmental stochasticity with no autocorrelation appeared statistically consistent with the data for all microcosm experiments (Fig. 2); however, when comparing model fits with the fpt AIC further model improvements were apparent.

The form of density dependence had a moderate effect on predictions, and removing density dependence greatly worsened predictions. We found that the Ricker model of density dependence minimised the ΔAIC value in simple communities though the Beverton–Holt also performed well (ΔAIC = 0.91), whereas in the complex communities the Gompertz model performed best overall (Table2). The undercompensatory dynamics displayed by the Gompertz model may be capturing the weak consumer–resource coupling in the complex community, whereas stronger compensation of the Ricker model better captured the strong coupling in the simple communities. Because the Gompertz model is less sensitive to density than the other models considered here, it may be a better model for populations embedded in complex systems where extrinsic factors play a significant role in regulation processes. Consistent with this interpretation, previous work has shown that concave forms of density dependence such as the Gompertz model are generally found in time series of natural populations (Sibly *et al*. 2005). In all microcosms, density-dependent models greatly outperformed the density-independent exponential growth model (Table2).

**Table 2:** ΔAIC values for each model and community type

Model 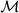	*k*	Simple community	Complex community
Ricker	4	**26.72**	400.86
Beverton–Holt	4	27.63	386.54
Logistic	4	79.92	271.38
Gompertz	4	68.93	**245.04**
Exponential	3	128.90	569.12
Log normal	4	45.81	**355.11**
Negative binomial	4	113.42	868.37
Gamma	4	**26.72**	400.86
No autocorrelation	4	26.72	400.86
AR	5	13.44	370.04
MA	5	**0.00**	21.76
ARMA	6	33.84	**0.00**
Demographic and environmental	4	**26.72**	400.86
No density dependence in variance	4	28.36	**397.45**
Environmental only	3	119.25	1231.23
Demographic only	3	37.20	1385.27

The number of parameters used per microcosm in the AIC calculation is given by *k* and bold numbers represent the best model within a set of comparisons. AIC, Akaike information criterion; AR, autoregressive; ARMA, autoregressive moving average; MA, moving average.

When using the Ricker model and assuming independently distributed observations with both demographic and environmental variability, the range of ΔAIC values among transition distributions had approximately the same magnitude as differences among density-dependence models. This suggests that the choice of transition distribution is a potentially important consideration when building models for extinction risk assessment, but one that is rarely tested. The gamma distribution predicted *τ*(*n*) best in the simple communities, whereas the log-normal was best in the complex communities (Table2). The weak performance of the negative binomial model relative to the continuous models implies that lattice effects may not be an important factor when modelling these populations.

When removing the assumption of independent error structures, MA models outperformed other models in the simple community experiment, whereas the ARMA model performed best in the complex community (Table2). As discussed in the Models sections, ARMA model terms measure the magnitude of inter- and intraspecific interactions and the tendency for interspecific interactions to dampen stochasticity. A negligible AR term in the simple community is indicative that inter- and intraspecific forces are balanced, whereas the presence of all negative AR terms in the complex communities indicates that intraspecific interactions are weaker than the intraspecific growth and interaction terms. The importance of the MA term in both simple and complex communities is indicative of weak regulation in the prey population and a corresponding tendency for perturbations in the predator population to be propagated by the prey population. Pure AR models performed relatively poorly in both community types, a surprising result due to the emphasis of previous work on this and similar models (e.g. Halley 1996; Cuddington & Yodzis 1999; Morales, 1999).

Comparing model variances, we found that demographic and environmental stochasticity were both important for predicting *τ*(*n*). Removing the density-dependent term in the demographic variance did not appear to lead to a difference in the simple communities (ΔAIC = 1.64), although it did in the complex communities (ΔAIC = 3.41). Interestingly, when looking at AIC values for the abundances (Appendix C, Table C1), rather than *τ*(*n*), our results are consistent with previous work that suggests that including the density dependence in demographic stochasticity is important (Drake 2005). The difference between *τ*(*n*)'s and abundances may be due to the fact that the density-dependent term in the demographic variance becomes approximately 1 at low abundances. For abundance time series, low population sizes are relatively rare and the estimates will tend to be dominated by the region where most abundance observations occur, whereas in the *τ*(*n*)'s low abundances will tend to have a larger relative effect because observations are more evenly dispersed across the range of observed abundances.

Finally, the results from the RMSE calculation (Table3) differed in some ways from the AIC values (Table2). This is likely due to the fact that the RMSE only considers the mean response of the predicted *τ*(*n*)'s rather than the whole distribution as in the AIC. Despite these differences, the overall best RMSE models were the same as the AIC comparison. The simple community had a RMSE in *τ*(*n*) of 19.58 days, predicted by the Ricker density-dependence model with demographic and environmental variability and a MA autocorrelation model. The minimum RMSE for the complex community was better than the simple communities at 5.91 days, predicted by the Ricker density-dependence model with demographic and environmental variability and an ARMA autocorrelation model. The inclusion of a suitable autocorrelation model improved the overall RMSE by about 20% in the simple community and by about 30% in the complex community.

**Table 3:** Root mean square error for each model and community type

Model 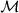	Simple community	Complex community
Ricker	23.23	7.84
Beverton–Holt	**22.51**	**7.18**
Logistic	26.38	8.73
Gompertz	23.11	**7.18**
Exponential	36.91	13.05
Log normal	**22.09**	8.44
Negative binomial	23.66	12.07
Gamma	23.23	**7.84**
No autocorrelation	23.23	7.18
AR	23.28	8.02
MA	**19.58**	6.15
ARMA	20.93	**5.91**
Demographic and environmental	**23.23**	7.84
No density dependence in variance	23.30	**7.74**
Environmental only	23.46	12.23
Demographic only	27.71	8.18

Bold numbers represent the best model within a set of comparisons. AR, autoregressive; ARMA, autoregressive moving average; MA, moving average.

### Measurement error

We tested the impact of a binomial sampling model on MA parameter estimates with data simulated from the simple community experiment. We found that the magnitudes of the MA parameter estimates due to measurement error were less (mean, −0.04, −0.04, −0.06) than the MLE estimates of the MA terms in the simple community experiment microcosms [mean(SE), −0.23(0.012), −0.48(0.017), −0.11(0.015)]. These results suggest that the MA terms present in the data are not due to sampling error and that another mechanism was likely responsible.

### Moving-average model dynamics

**Figure 3:**
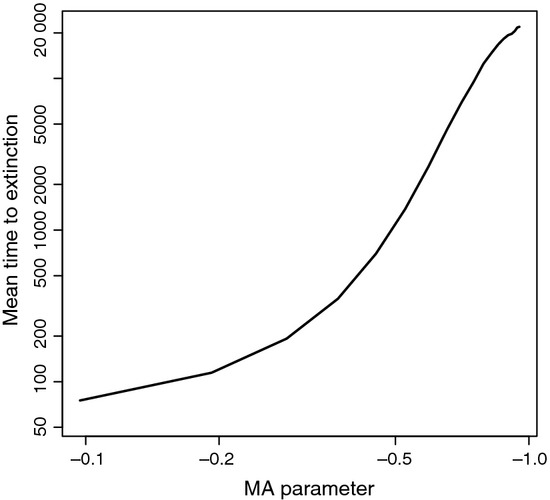
A log–log plot of the affect of a changing moving-average (MA) parameter value on the mean time to extinction. All parameters other than MA term were values estimated from microcosm 1.

We found that incorporating MA models into population growth processes can dramatically affect the MTE of a species (Fig. 3). As the magnitude of the MA parameter increased, persistence increased over three orders of magnitude in a faster than power law relationship. These results are similar to previous simulation studies exploring the impact of AR models on population persistence (Cuddington & Yodzis 1999; Morales, 1999; Petchey 2000) having similar order of magnitude impacts on population persistence as previous studies on AR models.

Given the potential importance of MA models in predicting abundances it is worth further understanding the impacts of these models on dynamics. In our formulation, the moving-average affects the growth rate through the log-scale abundances, and the magnitude of MA components is proportional to magnitude of perturbations from the expected value of the one-step transitions. The contribution to the growth rate by the moving-average component can then be interpreted as a rescaling of the log ratio of the observed and expected population abundances, 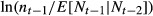 (Appendix C). When the population is higher than the expected value, a negative MA parameter (as found in all these microcosms) pulls the population towards the expected value, increasing the strength of population regulation (*sensu* Ziebarth *et al*. 2010) through a restorative force.

## Discussion

In this study, we assessed the effects in the predicted extinction risks of four different population modelling assumptions (the density-dependence form, the form of the transition pdf and autocorrelation in the growth rate and the nature of the process error variance model), but much remains to be done. Despite a well-developed body of literature describing the properties of population extinctions, very little has been done to quantitatively test these predictions (Griffen & Drake 2008). Here, we used experimental data to show that species interactions can be an important contributor to extinction risk while successfully testing the ability of autocorrelation models to account for these interactions. In addition, we demonstrate how and why many of the common assumptions associated with fitting population models to data can impact extinction risk predictions in real ecological systems. Our approach suggests that strong modelling tests – usually reserved only for simulation studies – coupled with detailed experimental data, can provide useful insights on modelling choices even in relatively simple systems.

Our efforts suggest that combining empirical and analytical methods can lead to a better understanding of the processes governing population dynamics. However, as suggested by Dennis & Taper (1994) the inference of ecological mechanisms must be treated with caution. Both Wolda (1991) and Dennis & Taper (1994) point out that it is impossible to distinguish ‘fluctuating equilibrium values’ from ‘fluctuating deviations from those equilibrium values’ using simple time series of abundance. We would attribute this variation to the environmental variance for both cases, though they may have different ecological and conservation implications. Despite these limitations, the models we explored here have the potential to incorporate extrinsic factors that can be used to explain variation in vital rates and improve predictive ability. However, this information needs to be both available and resolvable, which is not always the case. As shown by Knape & de Valpine (2010) even when time series of environmental variables are available, resolving these complex non-linear interactions is not a trivial task, although important exceptions do exist (e.g. Ponciano & Capistrán 2011). In more complex abundance models, even more difficulties exist. The process of distinguishing among mechanisms translates into uniquely identifying parameters from the available data. It is possible, and surprisingly easy to introduce parameter non-identifiability into stochastic models. Ponciano *et al*. (2012) show how data cloning can be used as a diagnostic tool to detect parameter non-identifiability. These complexities suggest that testing the inferential limitations of time-series abundance models for PVA will continue to be an important line of research.

Our first finding was that the form of density dependence for the same species differed depending on the community composition in which it was growing, in a way that is consistent with ecological theory. Thus, strong density dependence (overcompensatory dynamics), implied by the Ricker model, was found to be best in the simple community, whereas in the complex community a weaker form of density dependence, embodied by the Gompertz model, resulted in better predictions. The discrepancy in optimal density-dependent models for otherwise similar populations reflects community-level differences between a strongly coupled consumer–resource system and a more complex community with a number of biotic and abiotic interactions. This result implies that the best model for one population may simply not translate to other populations of the same species when ecological forces differ between communities (Murdoch & McCauley 1985), an assumption that is often made when a particular system lacks adequate data (e.g. Ferguson *et al*. 2012).

Although it is common practice to assume a log-normal transition distribution when fitting models to data of population growth, there are plausible biological reasons under which this distribution may not hold (Diserud & Engen 2000; Henson *et al*. 2001). We found that the gamma distribution did best in the simple community, whereas the log normal did best in the complex community. In addition, both demographic and environmental stochasticity were important for predictions in simple and complex microcosms. Predictions of quasi-extinction often only include an environmental variance term (e.g. Holmes *et al*. 2007), the dominant contribution at higher abundances. However, for populations truly at risk of extinction, our results suggest that this is not sufficient and that demographic and environmental models of stochasticity should be used.

An important open question for conservation and management is to identify when more complex multispecies process models can be used to improve abundance predictions (e.g. Sabo 2008). Our results show that accounting for interspecific interactions in PVA's may be possible in single-species time series through the use of appropriate autocorrelation models. The simplified statistical representation of species interactions that are provided by autocorrelation structures can improve predictions without the need for multispecies time-series data making this a powerful and tractable approach.

When facing the pressing need of a quantitative assessment of extinction risk, modellers and scientists rely by necessity on a number of model assumptions and simplifications. Choosing which simplifications and assumptions should be retained or else, discarded, seems to be a key component of a modeller's *savoir faire* that all too often is taken for granted. As put by Taper *et al*. (2008), models carry the meaning of science, and this puts a tremendous burden on the process of model selection. In that sense, our study represents one of the first examples we are aware of that illustrates why the structural adequacy (*sensu* Taper *et al*. (2008)) of ecological models should be routinely and extensively explored. We hope that our study represents a starting point for future explorations of the variance scaling and decomposition of population dynamics models with the goal of improving quantitative predictions in conservation and management.
