# Supplementary material for: Predicting the process of extinction in experimental microcosms and accounting for interspecific interactions in single-species time series
Source: Ecol Lett. 2013 Dec 5;17(2):251–9. doi: 10.1111/ele.12227 (PMC3912915; doi:10.1111/ele.12227)
Supplement: Supplementary file 1 [file ele0017-0251-sd1.pdf]

Appendix A: Derivation of mean and variance for abundances

Here we derive the mean and variance of population abundances using a stochastic process approach similar to previous work by Engen *et al.* (1998) and Melbourne & Hastings (2008). Let the offspring for individual i in a population be given by X_i. We assume that $E[X_i] = \lambda$ and $V[X_i] = \phi(\lambda)^2$. The number of individuals born for the next time step of a population with abundance N_{t-1} is given by $Y_t = \sum_{i=1}^{N_{t-1}} X_i$, where the X_i's are identically and independently distributed. It follows that $E[Y_t|N_{t-1} = n_{t-1}] = \lambda n_{t-1}$ and $V[Y_t|N_{t-1}] = n_{t-1}\phi(\lambda)^2$. The survival of offspring is given by $p_t = p_0 p(n_t)$, where p_0 is the density-independent component of survival and $p(n_t)$ is the density-dependent component of survival. The number of survivors out of the Y_t born is given by N_t and we assume a binomial distribution of survival, $(N_t \mid N_{t-1} = n_{t-1}, Y_t) \sim \text{BIN}(Y_t, p_{t-1})$. This variation in births and survival is the demographic variability.

We can use the properties of expected values to average over the unobserved random variable Y_t to get the next generations abundances,

$$\begin{aligned} E[N_t \mid N_{t-1} = n_{t-1}] &= E\left[E[N_t \mid N_{t-1} = n_{t-1}, Y_t]\right] \\ &= E\left[Y_t p_{t-1}\right] \\ &= \lambda n_{t-1} p_{t-1}. \end{aligned} \tag{A1}$$

Similarly, for the variance,

$$
\begin{aligned}
V[N_t \mid N_{t-1} = n_{t-1}] &= E[V[N_t \mid N_{t-1} = n_{t-1}, Y_t]] + V[E[N_t \mid N_{t-1} = n_{t-1}, Y_t]] \\
&= E[Y_t p_{t-1}(1 - p_{t-1})] + V[Y_t p_{t-1}] \\
&= E[Y_t] p_{t-1}(1 - p_{t-1}) + p_{t-1}^2 V[Y_t] \\
&= \lambda n_{t-1} p_{t-1}(1 - p_{t-1}) + p_{t-1}^2 n_{t-1} \phi(\lambda)^2 \\
&= \lambda n_{t-1} p_{t-1} + n_{t-1} p_{t-1}^2 (\phi(\lambda)^2 - \lambda). \qquad \text{(A2)}
\end{aligned}
$$

The density dependence can also be placed in the fecundity term, with survival as a constant, leading to a similar form for the mean and variance terms. It is also important to note that the terms λ and p_0 are not identifiable under the Poisson assumption for demographic stochasticity, $\phi(\lambda)^2 = \lambda$. Under the Poisson assumption $V[N_t \mid N_{t-1} = n_{t-1}] = \lambda n_{t-1} p_{t-1} = \lambda p_0 n_{t-1} p(n_{t-1})$. Therefore, we explored whether the parameter p_0 was identifiable in our data when the Poisson assumption is relaxed by fitting the joint-likelihood profile of p_0 and λ for the first microcosm experimental dataset (the likelihoods are fully described in Appendix C). As in the main manuscript we let the demographic stochasticity in reproduction be a constant, $\phi(\lambda)^2 = \phi^2$. We found that λ and p_0 were collinear (Figure A1) showing that it is not possible to jointly estimate both parameters from abundance data. Therefore we follow Melbourne & Hastings (2008) and treat λp_0 as a single parameter r. This leads to the following approximation for the demographic variance which ignores the density independent contribution in the binomial variance term:

$$
V[N_t \mid N_{t-1} = n_{t-1}] \approx r n_{t-1} p(n_{t-1}) + n_{t-1} p(n_{t-1})^2 (\phi(\lambda)^2 - r). \qquad \text{(A3)}
$$

This approximation can lead to estimates that predict higher or lower variance than the true value depending on the abundance and the underlying parameter values, however, simulations indicate that the approximation is reasonable over a range of values for p_0.

We included random environmental contributions in reproduction by allowing r to vary from year to year. This is done by defining W_t to be the distribution of the population's birth rate, where the birth rate can now vary from year to year with a mean λ and variance φ^2. Then $E[X_i \mid N_{t-1} = n_{t-1}, W_t = w_t] = w_t$ and $V[X_i \mid N_{t-1} = n_{t-1}, W_t = w_t] = \phi(w_t)^2$, while $E[Y_i \mid N_{t-1} = n_{t-1}, W_t = w_t] = w_t n_{t-1}$ and $V[Y_i \mid N_{t-1} = n_{t-1}, W_t = w_t] = n_{t-1}\phi(w_t)^2$. As above, assuming that survival is given by a conditional binomial distribution, $(N_t | N_{t-1} = n_{t-1},\, Y_t,\, W_t = w_t) \sim \mathrm{BIN}(Y_t,\, p_{t-1})$, now

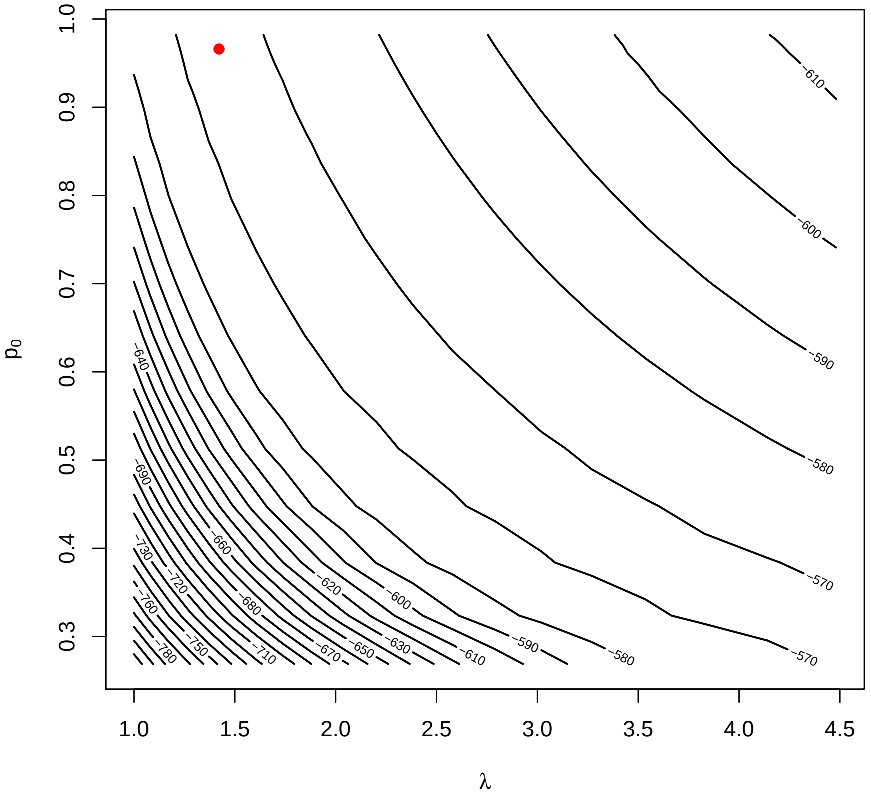

Figure A1: Contour likelihood of the mean (equation A1) and variance (equation A2) assuming a lognormal transition distribution. The maximum likelihood estimate is labeled with the red point. The lack of a well defined peak indicates that the parameters r and p_0 are collinear and are not independently identifiable.

the mean and variance conditional on the state of the environment, W_t, are:

$$E[N_t \mid N_{t-1} = n_{t-1}, W_t = w_t] = w_t n_{t-1} p(n_{t-1})$$

$$V[N_t \mid N_{t-1} = n_{t-1}, W_t = w_t] = w_t \left[p(n_{t-1})(1 - p(n_{t-1})) + n_{t-1} p(n_{t-1})^2 \phi(w_t)^2 \right].$$

Finally, averaging over the environmental state W_t gives a model with both demographic and environmental stochasticity. The mean is given by

$$
\begin{aligned}
E[N_t \mid N_{t-1} = n_{t-1}] &= E[E[N_t|N_{t-1}, W_t]] \\
&= E[W_t] n_{t-1} p_{t-1} \\
&= r n_{t-1} p(n_{t-1}),
\end{aligned}
$$

and the variance by

$$
\begin{aligned}
V[N_t \mid N_{t-1} = n_{t-1}] &= E[V[N_t|N_{t-1}, W_t]] + V[E[N_t|N_{t-1}, W_t]] \\
&= n_{t-1} E[W_t] p_{t-1}(1 - p_{t-1}) + n_{t-1} E[\phi(W_t)^2] p_{t-1}^2 + n_{t-1}^2 p_{t-1}^2 V[W_t] \\
&\approx n_{t-1} \left[r p(n_{t-1})(1 - p(n_{t-1})) + p(n_{t-1})^2 E[\phi(W_t)^2] \right] + n_{t-1}^2 p(n_{t-1})^2 \varphi^2, \quad \text{(A4)}
\end{aligned}
$$

where we used the approximation for the demographic term described above.

For estimation purposes in the manuscript, we assumed that the demographic variance in reproduction is proportional to the yearly realized reproductive value, $V[X_i|W_t = w_t] = \pi w_t$. For the term $E[\phi(W_t)^2]$ in equation A4, we can then plugin $\pi\lambda$. The variance is then given by,

$$
\begin{aligned}
V[N_t \mid N_{t-1} = n_{t-1}] &= n_{t-1} E[W_t] p_{t-1}(1 - p_{t-1}) + n_{t-1} E[\phi(W_t)^2] p_{t-1}^2 + n_{t-1}^2 p_{t-1}^2 V[W_t] \\
&\approx n_{t-1} r p(n_{t-1})(1 - p(n_{t-1})) + n_{t-1} \pi r p(n_{t-1})^2 + n_{t-1}^2 p(n_{t-1})^2 \varphi^2,
\end{aligned}
$$

where we have used the approximation due to the nonidentifiability of the density independent survival that was used above. Additionally, π and r in the demographic stochasticity are non-identifiable. Therefore we define the term, $\phi^2 \equiv r\pi$. We then get the form of the variance used in the manuscript,

$$
V[N_t \mid N_{t-1} = n_{t-1}] = n_{t-1} \left[r p(n_{t-1})(1 - p(n_{t-1})) + p(n_{t-1})^2 \phi^2 \right] + \varphi^2 n_{t-1}^2 p(n_{t-1})^2. \quad \text{(A5)}
$$

Appendix B: ARMA models

The dynamics of a single species in a linear interacting system can be re-expressed through a combination of lagged observations of the population abundance and lagged error terms (Royama, 1981). Here we show how to obtain these dynamics in a two-species system by substituting the dynamics of one species into the other. Beginning with the stochastic discrete-time Gompertz model, $N(t) = N(t-1)\exp[c + a\ln N(t-1) + \varepsilon(t)]$, we can re-express the dynamics as a linear function with the substitution $X(t) = \ln N(t)$ then take the natural log of both sides of the Gompertz equation. This gives $X(t) = X(t-1) + c + aX(t-1) + \varepsilon(t)$ or $X(t) = c + bX(t-1) + \varepsilon(t)$ were $b = 1 + a$. The strength of density dependence is then given by the parameter a which for a stationary process can go between -1 and 1, with $a = 0$ corresponding to a random walk. The range for the parameter b is then given by $0 \leq b \leq 2$. Consider a linear interacting stochastic system with this formulation, written as:

$$X_1(t) = c_1 + b_{11}X_1(t-1) + b_{12}X_2(t-1) + \varepsilon_1(t) \tag{B1}$$

$$X_2(t) = c_2 + b_{21}X_1(t-1) + b_{22}X_2(t-1) + \varepsilon_2(t) \tag{B2}$$

The error terms are independent nonidentical-normal distributions such that: $\varepsilon_n(t) \sim \text{Norm}(0, \sigma_n^2(t))$. We remove the constants c_1 and c_2 and reintroduce them later in order to simplify the algebra. Royama (1981) and Abbott *et al.* (2009) have shown that we can express the dynamics of $X_1(t)$ or $X_2(t)$ through a substitution process. The substitution for $X_1(t)$ is as follows, first solve equation (B1) for $X_2(t-1)$ in terms of the $X_1(t)$'s and plug this into equation (B2):

$$X_2(t-1) = \frac{1}{b_{12}}\left(X_1(t) - b_{11}X_1(t-1) - \varepsilon_1(t)\right)$$

$$X_2(t) = b_{21}X_1(t-1) + \frac{b_{22}}{b_{12}}\left(X_1(t-1) - b_{11}X_1(t-2) - \varepsilon_1(t-1)\right) + \varepsilon_2(t-1)$$

Note that this procedure implies that $b_{12} \neq 0$ or else there is no solution. Now, plugging this result back into equation (B1), then collecting terms gives

$$X_1(t) = b_{11}X_1(t-1) + b_{12}\left(b_{21}X_1(t-1) + \frac{b_{22}}{b_{12}}\left(X_1(t-1) - b_{11}X_1(t-2) - \varepsilon_1(t-1)\right) + \varepsilon_2(t-1)\right) + \varepsilon_1(t)$$

$$X_1(t) = \underbrace{(b_{11} + b_{22})X_1(t-1)}_{\text{density dependence}} + \underbrace{(b_{12}b_{21} - b_{11}b_{22})X_1(t-2)}_{AR} + \underbrace{\varepsilon_1(t) - b_{22}\varepsilon_1(t-1) + b_{12}\varepsilon_2(t)}_{MA}, \tag{B3}$$

where each model component is labeled in equation B3. If we include constant growth terms then the constant growth rate, $c_1(1-b_{22}) + c_2b_{12}$, needs to be added on to equation B3. The error term can be rewritten as a moving average process, but it can be seen immediately from equation B3 that the moving average parameter is controlled by $-b_{22}$, which propogates the error in species 1 at the previous time steps, $\varepsilon_1(t-1)$, back into $X_1(t)$, while a strong interaction term b_{12} propogates variability from species 2 at time t, which tends to move the moving average towards 0. Using the result that the sum of two independent moving average processes is another moving average process whose order is the same as that of the highest order of the component processes (result from Box & Jenkins (1970)), the process can be rewritten as:

$$\varepsilon_1(t) - b_{22}\varepsilon_1(t-1) + b_{12}\varepsilon_2(t) = \xi(t) + \theta(t)\xi(t-1). \tag{B4}$$

The right hand side of equation B4 is a new moving average process, ξ, with unknown moving average parameter, $\theta(t)$ and variance, $\sigma^2_\xi(t-1)$. The variance and autocovariance of left-hand side of equation B4 are known and given by

$$\gamma_0 = (1 + b_{22}^2)\sigma_1^2(t) + b_{12}\sigma_2^2(t)$$
$$\gamma_1 = -b_{22}\sigma_1^2(t-1),$$

while the variance and autocovariance of the right hand side is given by

$$\gamma_0 = (1 + \theta(t)^2)\sigma_\xi^2(t)$$
$$\gamma_1 = -\theta(t)\sigma_\xi^2(t-1).$$

We can then equate the autocovariances to give

$$-\theta(t)\sigma_\xi^2(t-1) = -b_{22}\sigma_1^2(t-1)$$
$$\sigma_\xi^2(t-1) = \frac{b_{22}}{\theta(t)}\sigma_1^2(t-1).$$

Plugging this in for $\sigma_\xi^2(t)$ in the variances,

$$(1+b_{22}^2)\sigma_1^2(t) + b_{12}\sigma_2^2(t) = (1+\theta(t)^2)\sigma_\xi^2(t)$$
$$(1+b_{22}^2)\sigma_1^2(t) + b_{12}\sigma_2^2(t) = (1+\theta(t)^2)\frac{b_{22}}{\theta(t)}\sigma_1^2(t)$$
$$\theta(t)\left[(1+b_{22}^2)\sigma_1^2(t) + b_{12}\sigma_2^2(t)\right] = (1+\theta(t)^2)b_{22}\sigma_1^2(t)$$
$$\theta(t)\left[(1+b_{22}^2)\sigma_1^2(t) + b_{12}\sigma_2^2(t)\right] = (1+\theta(t)^2)b_{22}\sigma_1^2(t)$$
$$-\theta(t)^2 b_{22}\sigma_1^2(t) + \theta(t)\left[(1+b_{22}^2)\sigma_1^2(t) + b_{12}\sigma_2^2(t)\right] - b_{22}\sigma_1^2(t) = 0$$

We can then solve for $\theta(t)$ using the quadratic formula,

$$\theta(t) = \frac{-B(t) \pm \sqrt{B(t)^2 - 4(b_{22}\sigma_1^2(t))^2}}{-2b_{22}\sigma_1^2(t)}, \tag{B5}$$

where $B(t) = (1+b_{22}^2)\sigma_1^2(t) + b_{12}\sigma_2^2(t)$. For estimation purposes we assume $\theta(t) = \theta$. This is likely to be a reasonable assumption when demographic stochasticity is low but becomes worse as it increases. A time-dependency also enters the variance of the process $\sigma_\xi^2(t)$ which is also dependent on $\sigma_1^2(t)$ and $\theta(t)$. When using a form of density dependence other than the Gompertz the interaction equations are no longer linear. However, the Ricker model was studied by Abbott *et al.* (2009) who found that estimated number of AR and MA terms was consistent in model simulations. Based on our these assumptions we apply this model as a rough, but potentially useful, approximation to the complex stochastic dynamics that can be induced through predator-prey interactions.

Appendix C: Likelihoods

We fit gamma, log-normal, and negative binomial distributions to the data by setting mean and variance for the distributions to expressions derived in Appendix A. Here we use some shortcut notation for convenience,

$$
\begin{aligned}
\mathrm{E}[N_t|N_{t-1}=n_{t-1},\, N_{t-2}=n_{t-2}] &\equiv \mu_t = r n_{t-1} p(n_{t-1},\, n_{t-2}) && \text{(C6a)}\\
\mathrm{Var}[N_t|N_{t-1}=n_{t-1},\, N_{t-2}=n_{t-2}] &\equiv \sigma_t^2 = n_{t-1}\left[p(n_{t-1},n_{t-2})(1-p(n_{t-1},n_{t-2}))r + \right.\\
&\left. p(n_{t-1},n_{t-2})^2\phi^2\right] + \varphi^2 n_{t-1}^2 p(n_{t-1})^2. && \text{(C6b)}
\end{aligned}
$$

We fit all models by matching the mean and variance terms in (C6) to the distribution mean and variance. For the lognormal distribution this leads to:

$$
(N_t|N_{t-1},\, N_{t-2}) \sim \mathrm{LN}\left(\mu_{\ln} = \log(\mu_t) - \frac{1}{2}\sigma_{\ln}^2,\, \sigma_{\ln}^2 = \ln(\sigma_t^2/\mu_t^2 + 1)\right). \tag{C7}
$$

Similarly we fit the gamma distribution to the abundance transitions with shape (k) and scale (θ) parameters given by

$$
(N_t|N_{t-1},\, N_{t-2}) \sim \mathrm{Gamma}\left(k = \frac{\mu_t^2}{\sigma_t^2},\, \theta = \frac{\sigma_t^2}{\mu_t}\right).
$$

The negative binomial distribution was also fit to the abundance data. This model was parameterized by the mean (μ_{NB}) and size (k) parameters,

$$
(N_t|N_{t-1},\, N_{t-2}) \sim \mathrm{NBin}\left(\mu_{\mathrm{NB}} = \mu_t,\, k = \frac{\varphi^2}{r^2}\right).
$$

In the negative binomial the demographic variance is assumed to be equal to the growth rate, $\phi(\lambda)^2 = r$, while the size parameter, k, is related the environmental variance.

We used the one-step formulations of the AR, MA, and ARMA processes (Shumway & Stoffer, 2006). An exploratory analysis suggested that the autocorrelation processes operated on the scale of the population growth rate, $\ln\left(\frac{N_t}{n_{t-1}}\right)$, therefore we included the autocorrelation as

multiplicative processes such that they were additive to the density dependence on the log-scale. This analyses also suggested that log-population abundances performed better than abundances so all autocorrelation models are formulated with this transformation. All models contained an AR(1) contribution through population growth and regulation processes contained in $rn_{t-1}p(n_{t-1})$. The AR(2) process was modeled as a contribution to the population growth rate given by $\psi \ln(n_{t-2})$. The MA(1) process was also modeled as a contribution to the population growth rate, this term was modeled as, $\theta\left(\ln(n_{t-1}) - \ln(\mu_{t-1})\right)$. The mean of the process given in (C6) with the full ARMA process therefore becomes

$$\mu_t = rn_{t-1}p(n_{t-1})\exp[\psi \ln(n_{t-2}) + \theta(\ln(n_{t-1} - \mu_{t-1})]. \tag{C8}$$

The ARMA processes are additive combinations of the AR and MA components so we can remove the AR(2) term by setting $\psi = 0$ and remove the MA(1) term by setting $\theta = 0$ to examine models nested to the ARMA(2,1). Although even higher order ARMA processes can be fit to the data using similar methods, we limited ourselves to ARMA(2,1) processes due to the patterns observed in the empirical autocorrelation function of the population growth rate (Figure C2).

The likelihoods of the abundances for these data were calculated using the transition probabilities with the transition distributions defined above:

$$L(\theta; N_t) = \prod_{t=3}^{t_{max}} P(N_t | N_{t-1},\, N_{t-2}; \Theta). \tag{C9}$$

Here, Θ is the set of parameters to optimize over, and t iterates over all observations up to the time of the final observation at t_{max}. For all models we condition on the first two observations so the actual number of transitions calculated is given by $t_{max} - 2$. As an example, consider fitting the Ricker model with an ARMA(2,1) autocorrelation structure and a lognormal transition distribution. The mean and variance for each time step given in (C6) are determined by setting $p(n_{t-1},\, n_{t-2}) = \exp\left[-bn_{t-1} + \psi \ln(n_{t-2}) + \theta(\ln(n_{t-1} - \mu_{t-1})\right]$, then plugging in the resulting values for μ_t and σ_t^2 into (C7) to get the one-step probabilities for all observed abundances except for the first two, which are being conditioned on. In this model the parameters to be optimized over are $\Theta = (r,\, b,\, \psi,\, \theta,\, \phi^2,\, \varphi^2)$. These probability values are plugged into (C9) to get the resulting likelihood of all the observations. We maximized the log-likelihood as a function Θ using the Rgenoud package

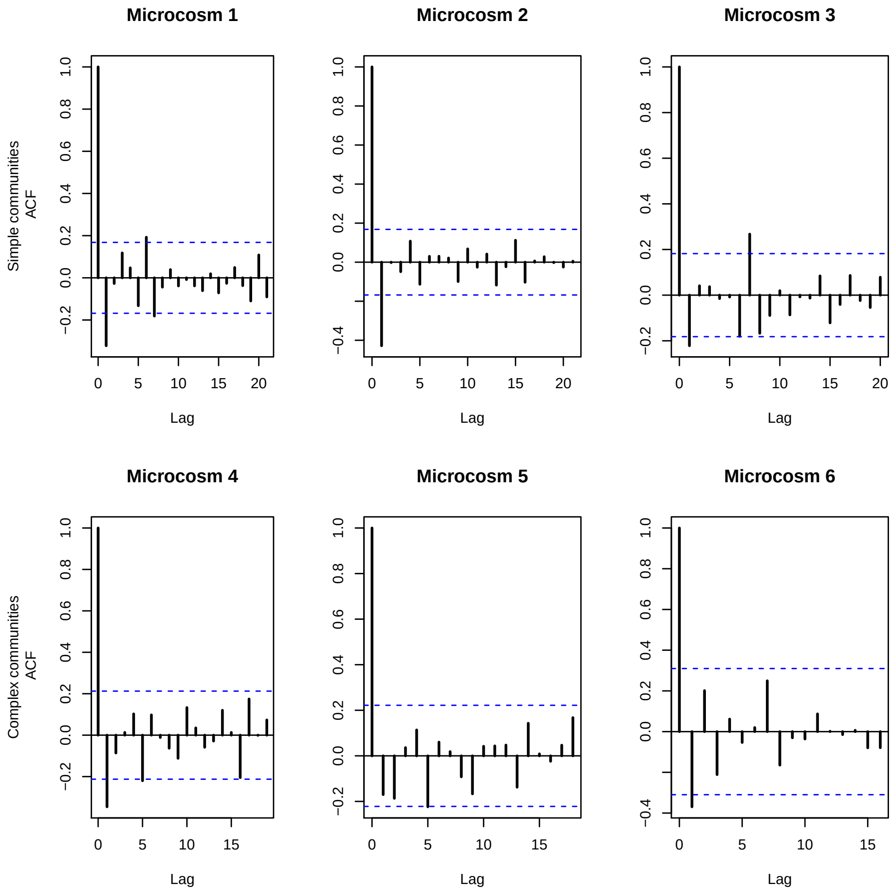

Figure C2: Dark bars are the empirically realized values for the autocorrelation at each lag. Horizontal blue line is the approximate 95% confidence interval.

(Mebane, Jr. & Sekhon, 2011) in R (R Development Core Team, 2012). We also note that the logistic form of density dependence, $p(n_{t-1}) = 1 - bn_{t-1}$, was constrained in the optimization by applying a constraint to the likelihood such that $0 < p(n_{t-1}) < 1$. The ΔAIC values for the abundances are presented in Table C1.

Table C1: ΔAIC values for each model fit to the population abundances by community type. Note - The number of parameters used in the AIC calculation is given by k and bold numbers represent the best model within a set of comparisons.

Model $\mathcal{M}$	k	Simple community	Complex community
Ricker	4	21.77	9.02
Beverton-Holt	4	14.32	8.15
Logistic	4	12.73	10.92
Gompertz	4	**3.62**	**5.76**
Exponential	3	66.91	16.97
Log-normal	4	**6.65**	10.41
Negative binomial	4	63.97	54.17
Gamma	4	21.77	**9.02**
MA	5	0.68	**0.00**
No correlation	4	21.77	9.02
ARMA	6	**0.00**	4.32
AR	5	17.81	8.78
Demographic and Environmental	4	**21.77**	**9.02**
No density dependence in variance	4	72.91	22.97
Environmental only	3	72.42	81.39
Demographic only	3	54.68	64.64

Bibliography

Abbott, K.C., Ripa, J. & Ives, A.R. (2009). Environmental variation in ecological communities and inferences from single-species data. *Ecology*, 90, 1268–1278.

Box, G. & Jenkins, G. (1970). *Time Series Analysis: Forecasting and Control.* Holden-Day, San Francisco.

Mebane, Jr., W.R. & Sekhon, J.S. (2011). Genetic optimization using derivatives: The rgenoud package for R. *Journal of Statistical Software*, 42, 1–26.

Engen, S., Bakke, O.y. & Islam, A. (1998). Demographic and environmental stochasticity-concepts and definitions. *Biometrics*, 54, 840–846.

Melbourne, B.A. & Hastings, A. (2008). Extinction risk depends strongly on factors contributing to stochasticity. *Nature*, 454, 100–103.

R Development Core Team (2012). R: A language and environment for statistical computing.

Shumway, R. & Stoffer, D. (2006). *Time series analysis and its applications.* Springer, New York.

Royama, T. (1981). Fundamental concepts and methodology for the analysis of animal population dynamics, with particular reference to univoltine species. *Ecol. Monogr.*, 51, 473–493.
